# Supplementary material for: Ultrasound-Guided Histotripsy Triggers the Release of Tumor-Associated Antigens from Breast Cancers
Source: Cancers (Basel). 2025 Jan 8;17(2):183. doi: 10.3390/cancers17020183 (PMC11764245; doi:10.3390/cancers17020183)
Supplement: Supplementary file 1 [file cancers-17-00183-s001.zip › cancers-3405837-supplementary.pdf]

Figure 5

A

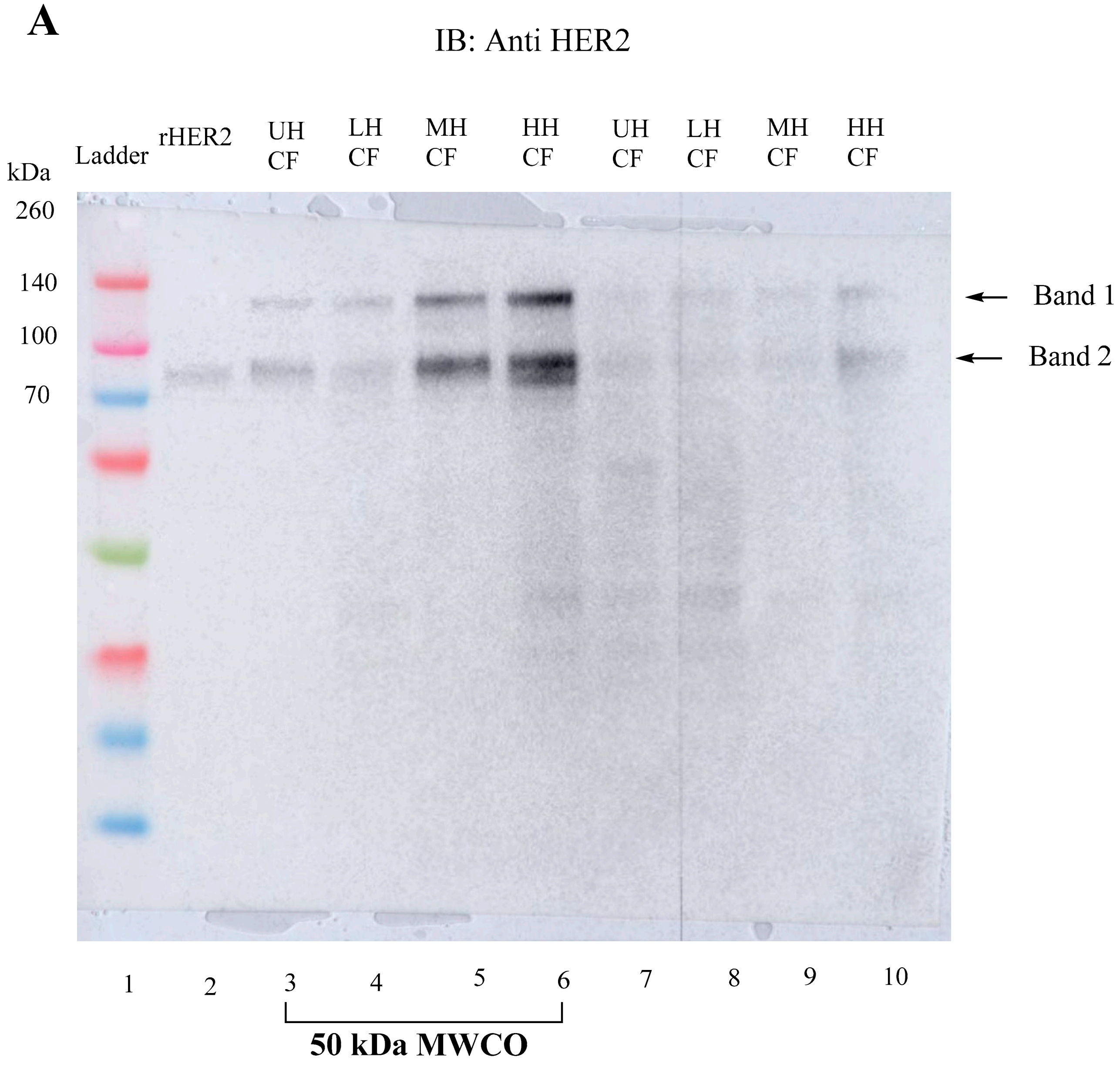

IB: Anti HER2
kDa
260
140
100
70
Ladder
rHER2
UH CF
LH CF
MH CF
HH CF
UH CF
LH CF
MH CF
HH CF
Band 1
Band 2
1
2
3
4
5
6
7
8
9
10
50 kDa MWCO

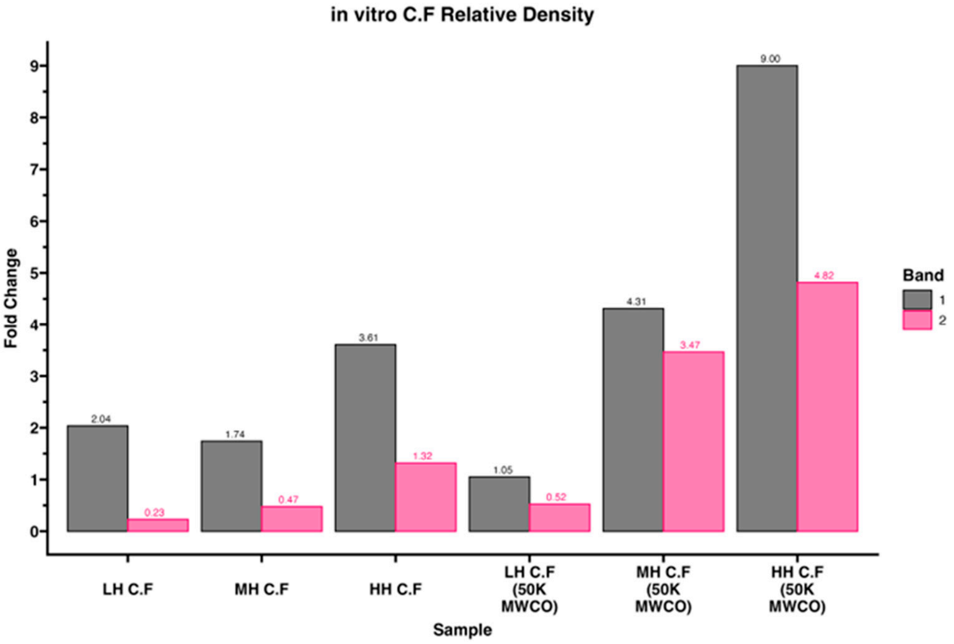
in vitro C.F Relative Density
Fold Change
Sample
Band
1
2
0
1
2
3
4
5
6
7
8
9
2.04
0.23
1.74
0.47
3.61
1.32
1.05
0.52
4.31
3.47
9.00
4.82
LH C.F
MH C.F
HH C.F
LH C.F (50K MWCO)
MH C.F (50K MWCO)
HH C.F (50K MWCO)

B

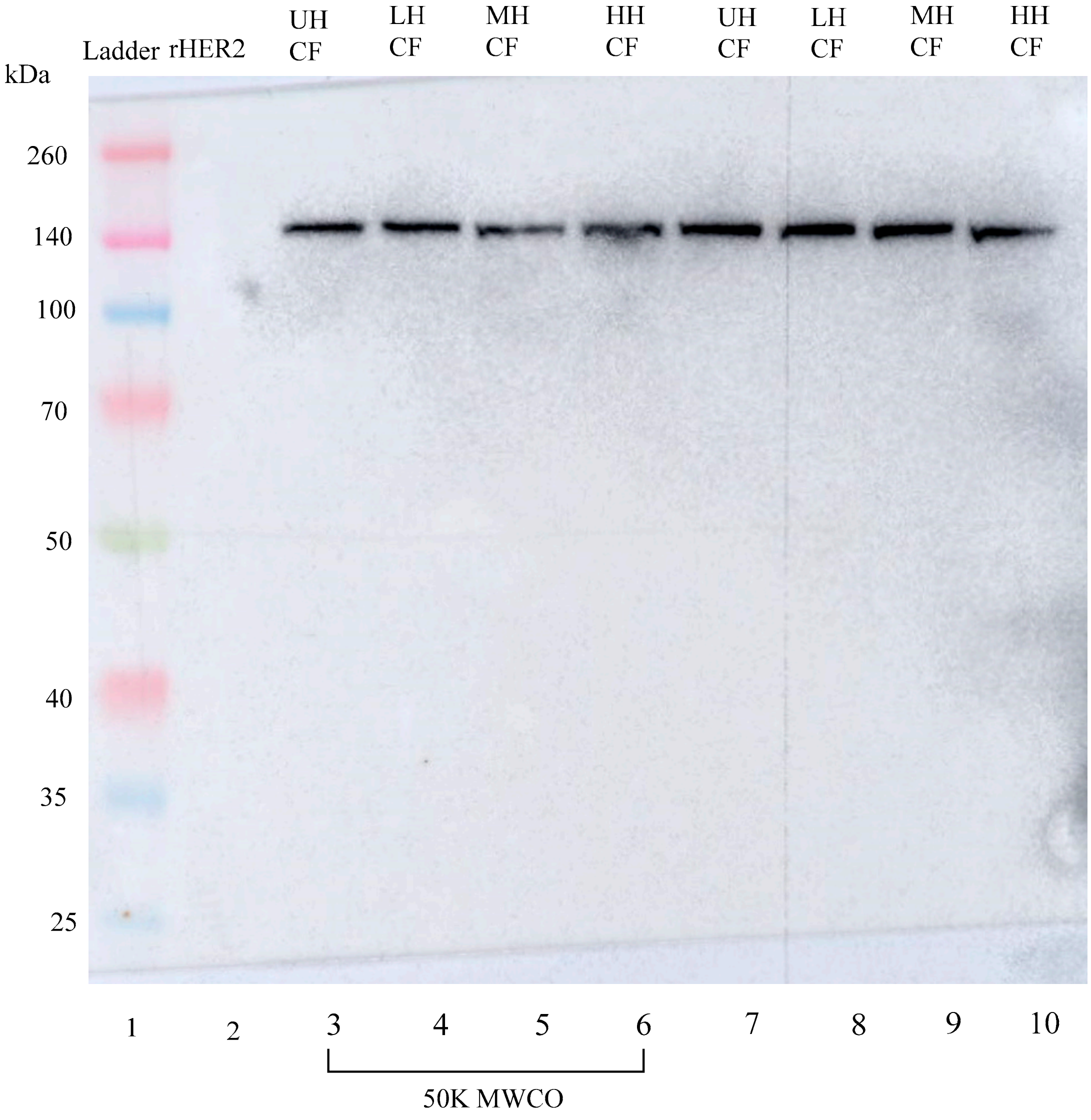

Figure S1. Original figures of Figure 5

Figure 6

A

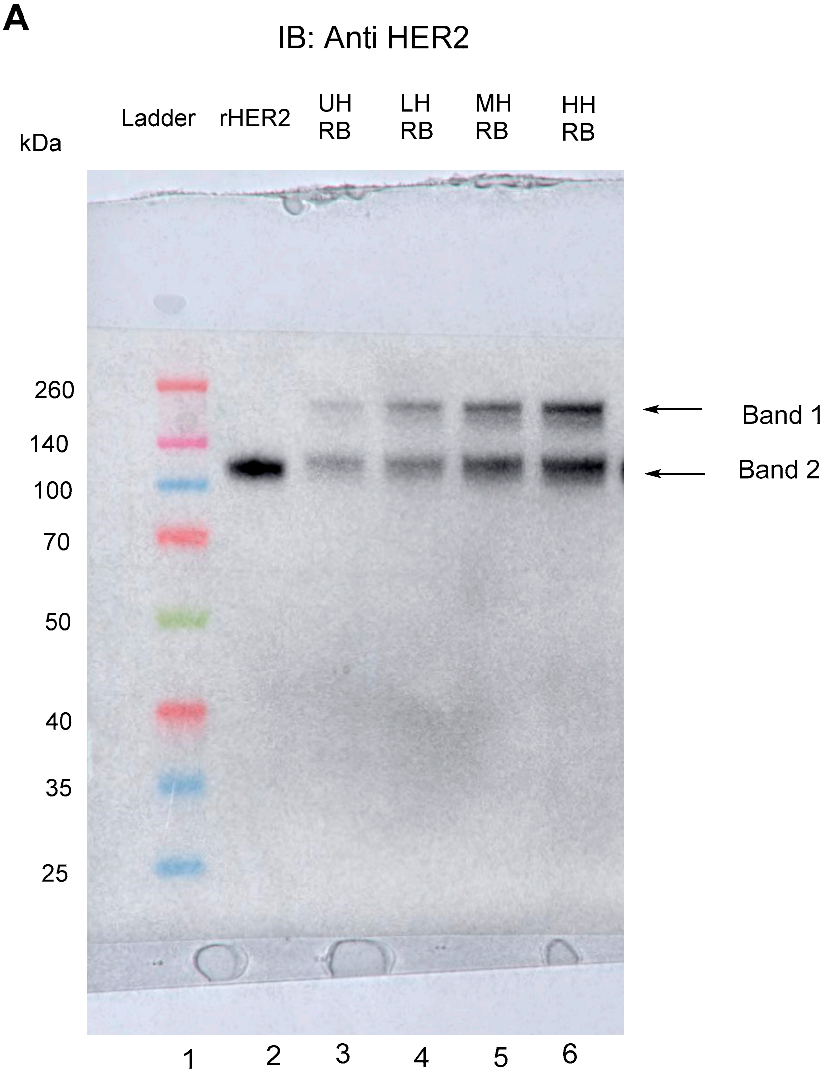

IB: Anti HER2
kDa
Ladder
rHER2
UH RB
LH RB
MH RB
HH RB
260
140
100
70
50
40
35
25
Band 1
Band 2
1
2
3
4
5
6

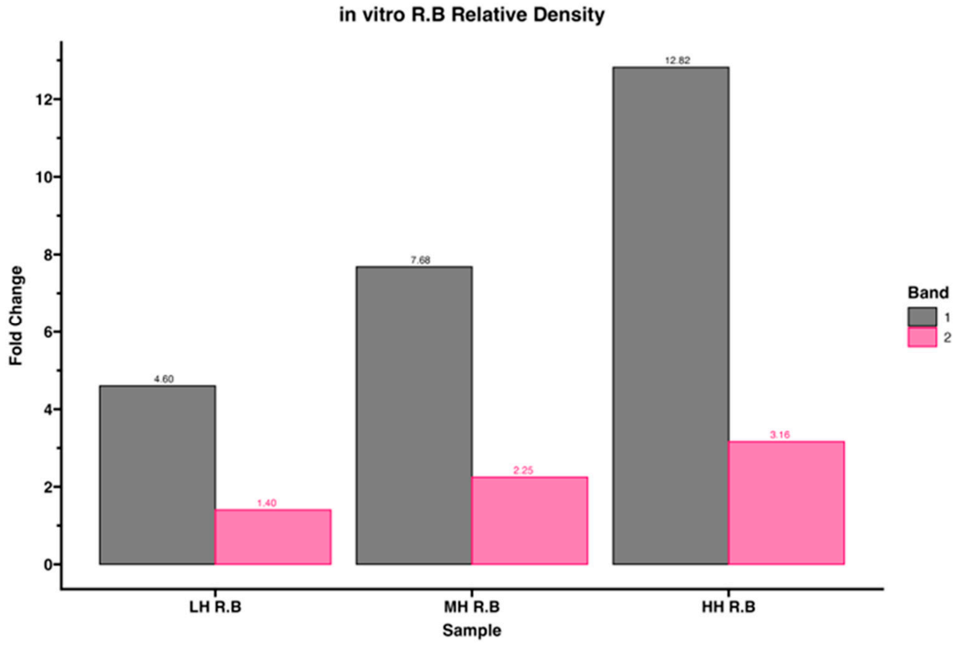

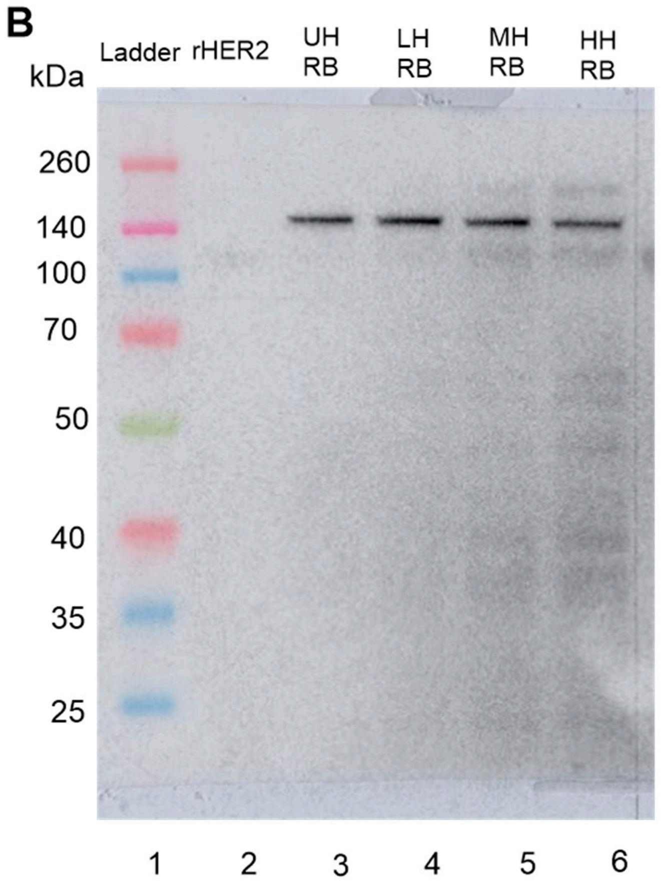

Figure S2. Original figures of Figure 6

Figure 9

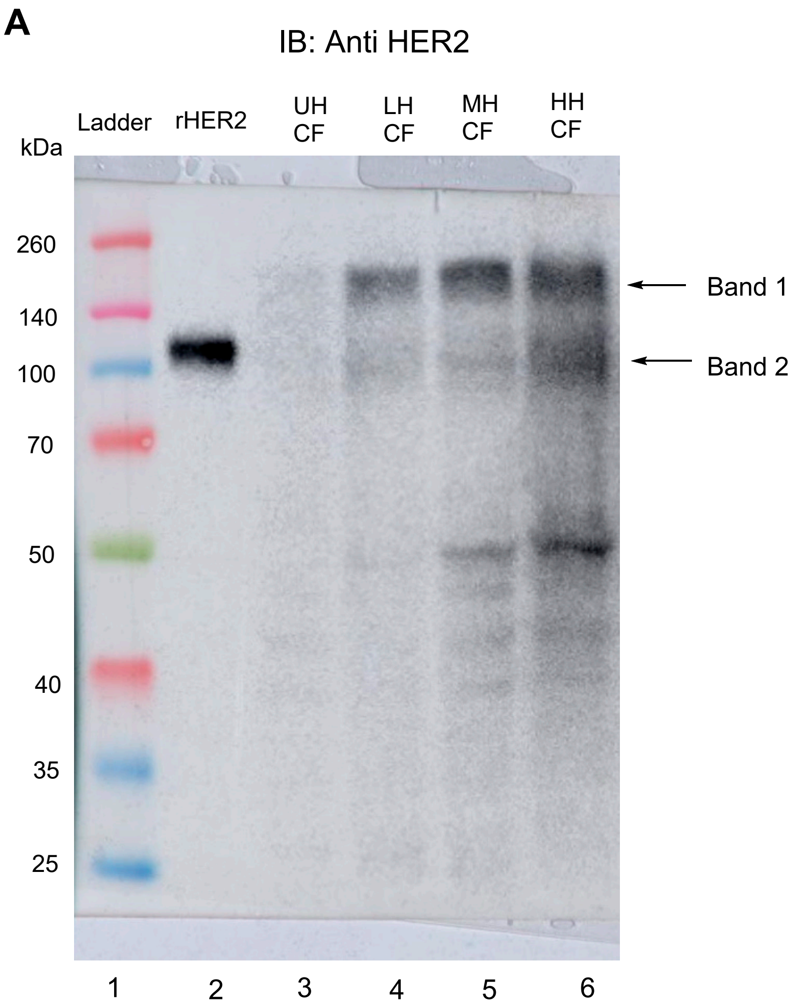
A
IB: Anti HER2
kDa
Ladder
rHER2
UH CF
LH CF
MH CF
HH CF
260
140
100
70
50
40
35
25
Band 1
Band 2
1
2
3
4
5
6

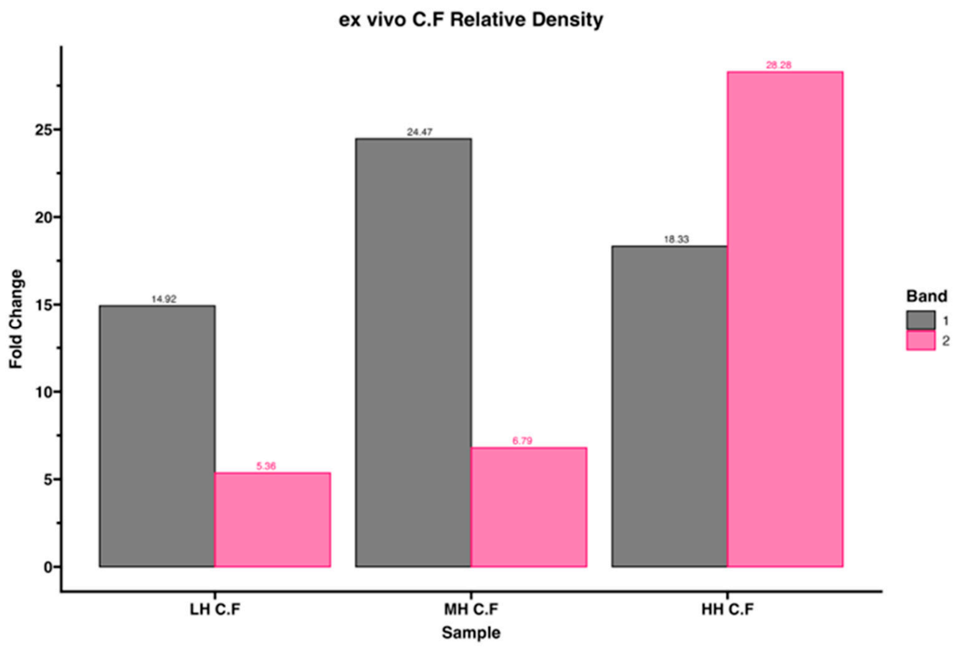
ex vivo C.F Relative Density
Fold Change
0
5
10
15
20
25
14.92
5.36
24.47
6.79
18.33
28.28
LH C.F
MH C.F
HH C.F
Sample
Band
1
2

B

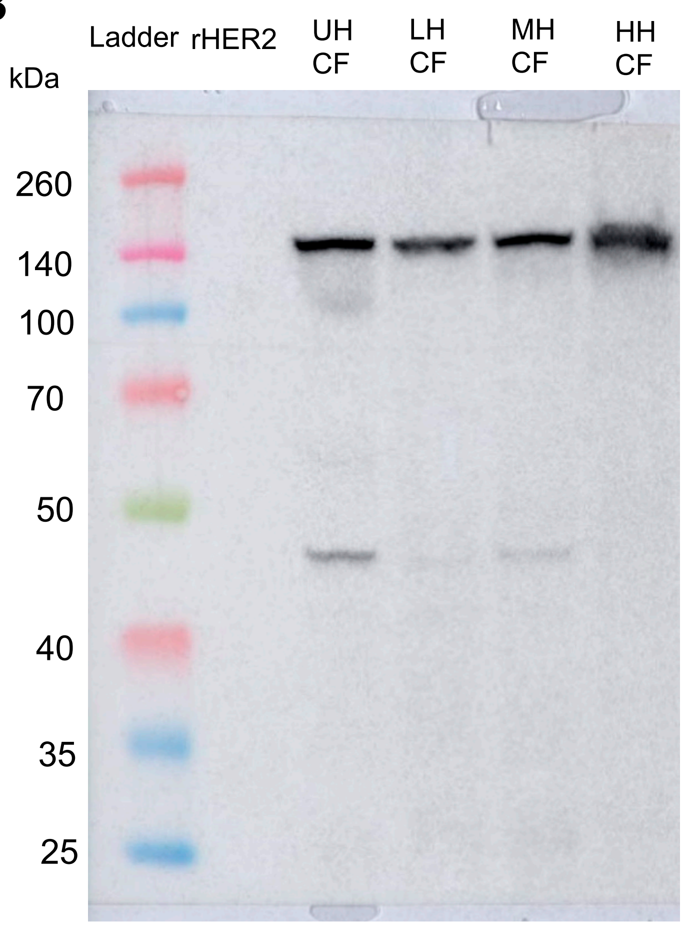
Ladder
rHER2
UH CF
LH CF
MH CF
HH CF
kDa
260
140
100
70
50
40
35
25

Figure S3. Original figures of Figure 9

Figure 10

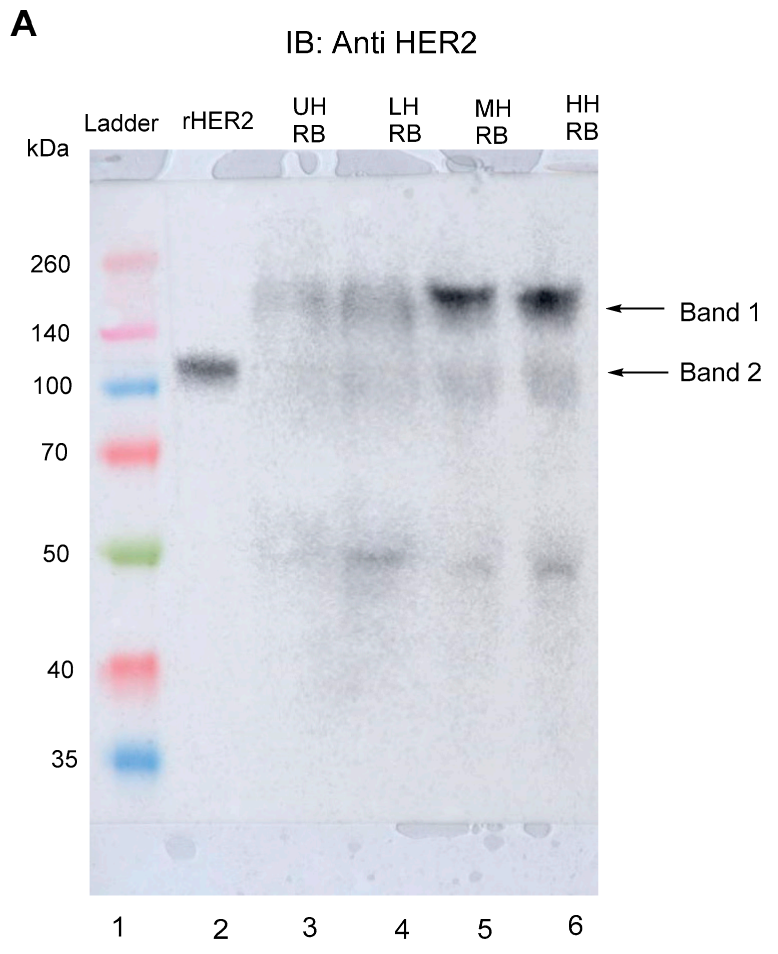

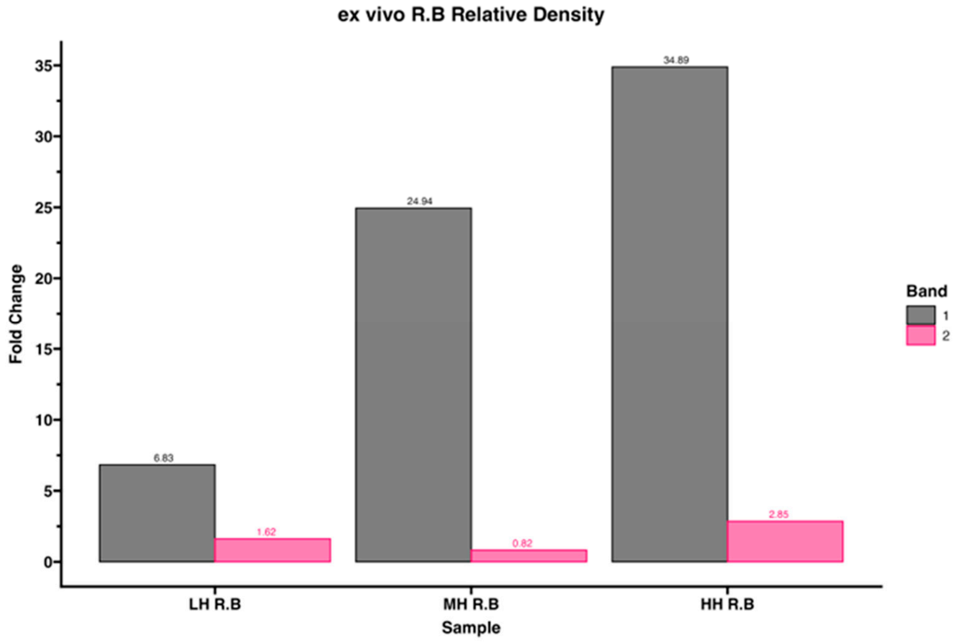

B

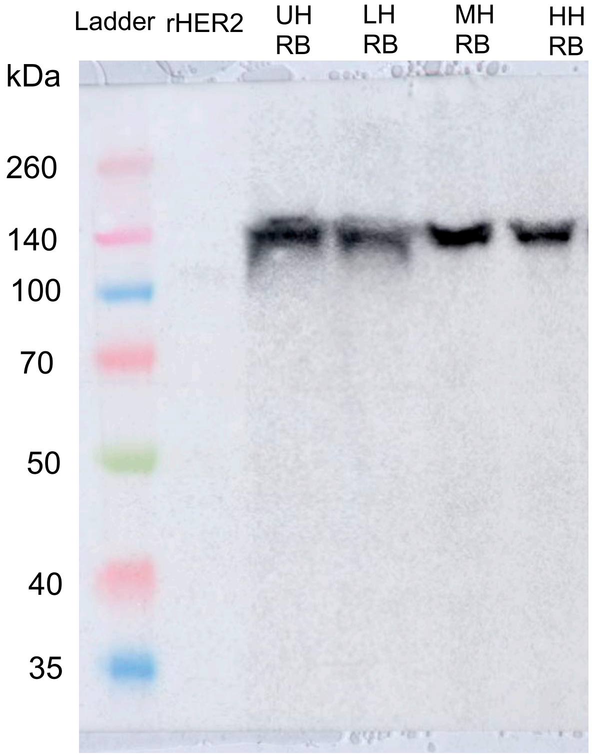

Figure S4. Original figures of Figure 10

Figure 11

E0771E2 tumor in vivo histotripsy west blot probed with monoclonal HER2 antibody

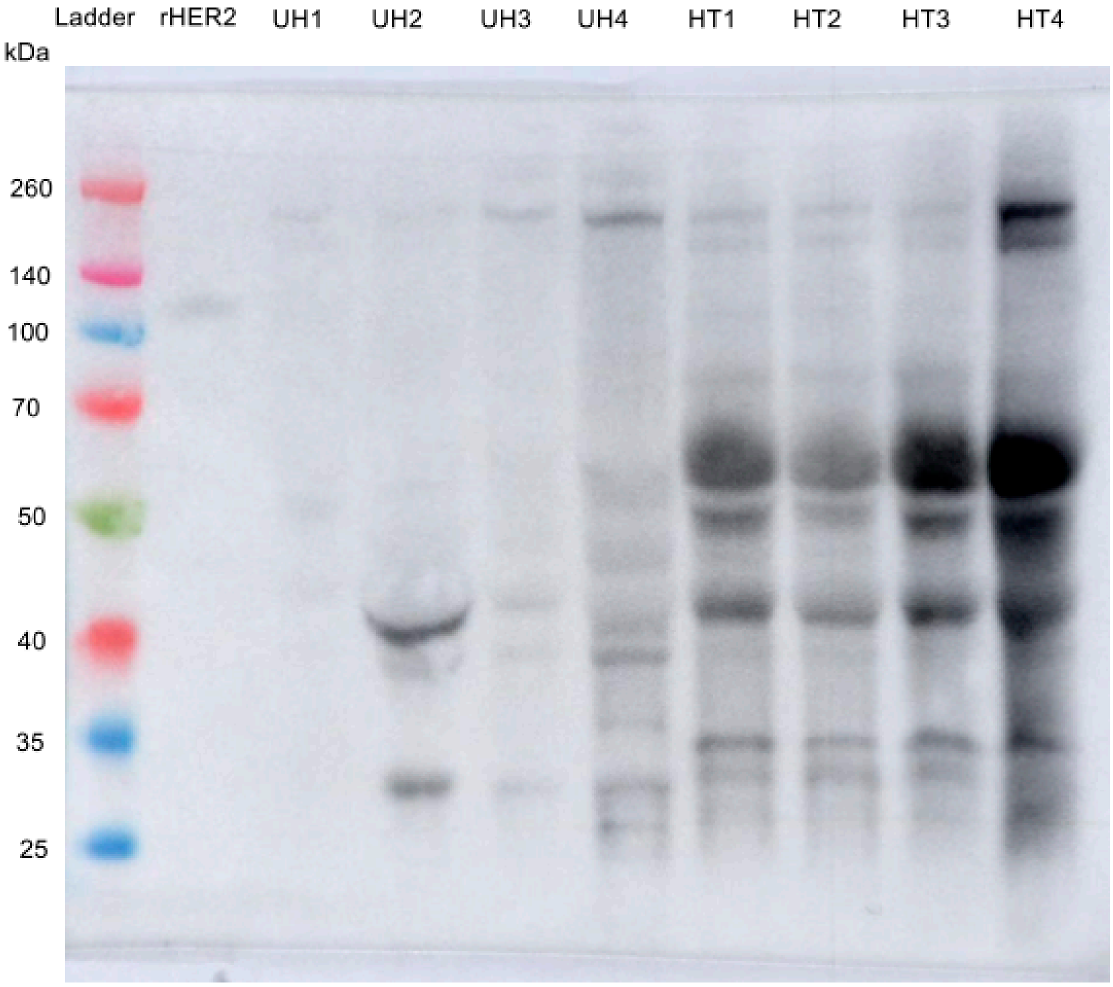

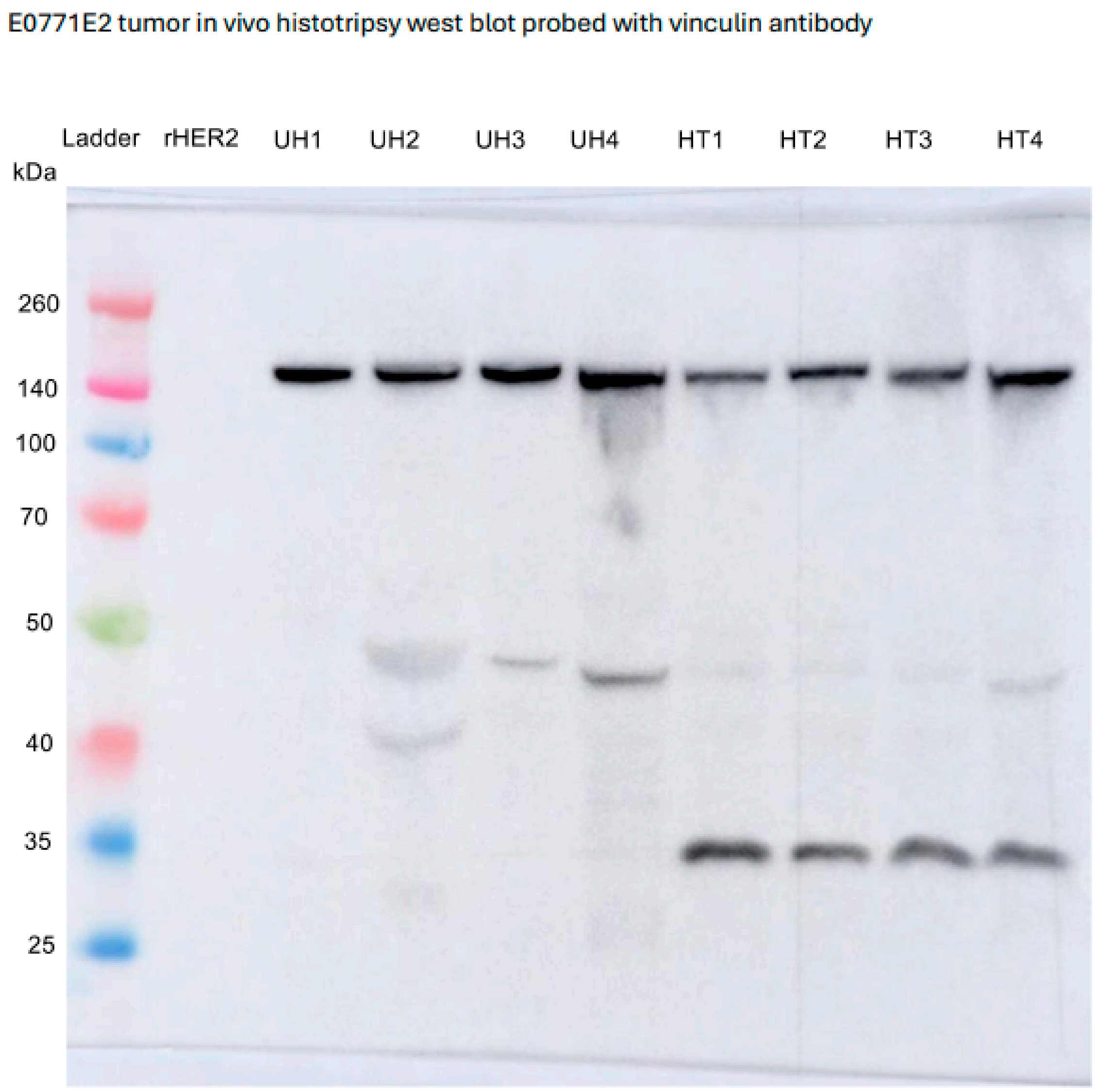

Figure S5. Original figures of Figure 11
